# Prediction of the Location of the Pyramidal Tract in Patients with Thalamic or Basal Ganglia Tumors

**DOI:** 10.1371/journal.pone.0048585

**Published:** 2012-11-14

**Authors:** YuanZheng Hou, XiaoLei Chen, BaiNan Xu

**Affiliations:** 1 Department of Neurosurgery, Chinese People's Liberation Army(PLA) General Hospital, Beijng, People's Republic of China; UCSF, United States of America

## Abstract

**Background:**

Locating the pyramidal tract (PT) is difficult in patients with thalamic or basal ganglia tumors, especially when the surrounding anatomical structures cannot be identified using computed tomography or magnetic resonance images. Hence, we objected to find a way to predict the location of the PT in patients with thalamic and basal ganglia tumors

**Methodology/Principal Findings:**

In 59 patents with thalamic or basal ganglia tumors, the PTs were constructed by with diffusion tensor imaging (DTI)-based fiber tracking (FT). In axial slices crossing the foramen of Monro, the tumor position was classified according to three lines. Line 1 was vertical and crossed the vertex point of the anterior limbs of the internal capsule. Lines 2 and line 3 were horizontal and crossed the foramen of Monro and joint of the middle and lateral thirds of the posterior limbs, respectively. Six (10.17%) patients were diagnosed with type 1 tumor, six (10.17%) with type 2, seven (11.86%) with type 3a, five (8.47%) with type 3b, 17 (28.81%) with type 4a, six (10.17%) with type 4b, three (5.08%) with type 5, and nine (15.25%) with type 6. In type 1 tumors, the PTs were located at the 12 o'clock position of the tumor, type 2 at six o'clock, type 3a between nine and 12 o'clock, type 3 between six and nine o'clock, type 4a between 12 and three o'clock, type 4b at three o'clock, type 5 between six and nine o'clock, and type 6 between three and six o'clock.

**Conclusions/Significance:**

The position of the PT relative to the tumor could be determined according to the tumor location. These results could prove helpful in determining the location of the PT preoperatively.

## Introduction

Thalamic and basal ganglia tumors account for approximately 1–5% of cerebral tumors and are frequently encountered in children and young adults [1], [2]. Historically, surgery has not been considered an option because of high morbidity and mortality due to their deep-seated location and surrounding vital structures [1]. Although surgery for thalamic and basal ganglia tumors has become much safer, it is still a challenge to protect normal function while maximizing the extent of tumor resection [3]. One of the structures that require protection is the pyramidal tract (PT). The PT, also known as the corticospinal tract, is required for the transmission of motor information associated with voluntary movements, and PT damage can result in muscle weakness, loss of muscle control, tremor, and paralysis on the contralateral side. The PT has a very close relationship with thalamic or basal ganglia tumors and is obviously displaced when these tumors are present. As a result, the relative positions of the PTs and tumors may vary greatly in different patients [4], [5]. Preoperatively, surgeons must determine the location of the PT according to the surrounding anatomical landmarks, based on experience and anatomical knowledge [6]. This method is not always effective, especially when the anatomical structures cannot be identified by computed tomography (CT) or magnetic resonance images (MRIs) owing to the space-occupying effect of the tumor or cerebral edema. Diffusion tensor imaging (DTI) and fiber tracking (FT) are currently the only methods to visualize the specific white matter tracts in vivo [7], [8]. Many authors have reported encouraging results in PT protection during surgery by integrating tractograms of the PT into a neuronavigation system [4], [5]. Moshel et al. reported preliminary experiences confirming the location of the PT preoperatively in patients with thalamic tumors. The authors concluded that tractograms were helpful in determining the location of the PTs and choosing the surgical approach [6]. However, this technique require expertise and proper software and is only available in certain major medical centers. Therefore, it is still necessary to find ways to assist the surgeon in judging the location of the PTs in thalamic and basal ganglia tumors. In this paper, we retrospectively collected patients with thalamic or basal ganglion tumors and investigated the relative position of the PT and tumor, by using DTI-based FT. We sought to determine the trend for the prediction of the location of the PT in patients with thalamic or basal ganglia tumors.

## Materials and Methods

### Ethics Statement

MRI scanning was approved by Medical Science Ethics Committee of General Hospital of the Chinese People's Liberation Army. Signed informed consent was provided by each patient or by an appropriate family member before MRI scan.

### Patient Population

We collected data retrospectively on patients with thalamic or basal ganglia tumors in our hospital from March 2009 to March 2011. All patients received MRI scans, and tractogram of the PT were constructed by DTI-based FT. If the PT was affected severely by the tumor or edema and the PTs could not be revealed by DTI-based FT, the patient was excluded. Patients were also excluded if tumors were found in other regions, such as the frontal, parietal and occipital lobe, because these tumors might demonstrate different and more complicated growth patterns.

### MRI and Data Processing

MRI scans were performed with a 1.5 Tesla scanner (Siemens Espree, Erlangen, Germany). The DTI data were acquired with single-shot spin-echo diffusion-weighted echo-planar sequence [echo time (TE) 147 ms, repetition time (TR) 9400 ms, matrix size 128×128, field of view (FOV) 251×251 mm, slice thickness 3 mm]. The diffusion-weighting (high b value) was 1000 s/mm2. This sequence is based on a balanced diffusion gradient design which strongly minimizes eddy-current artifacts compared to a single-refocused design [4]. For each patient, one null image (b0; low b value: 0 s/mm2) and 12 gradient directions were obtained with a voxel size of 1.9×1.9×3 mm. In total, 40 serial slices were measured in each patient. Applying five averages, the total DTI measurement required 10 min. Anatomical images were also obtained by T1-weighted 3D magnetization-prepared rapid acquisition gradient-echo sequence (TE 3.02 ms, TR 1650 ms, matrix size 256×256, FOV 250×250 mm, slice thickness 1 mm). Standard T2 and T2 fluid-attenuated inversion recovery (FLAIR) sequences were also scanned. The axial plane of all the sequences was in parallel with the imaginary line between the anterior and posterior commissure (AC-PC line).All the image data was imported into the planning software (iPlan 2.6, BrainLab, Feldkirchen, Germany) for further analysis after data conversion by PatXfer 5.1(BrainLab, BrainLab, Feldkirchen, Germany).

### Fiber Tracking

The diffusion weighted images was processed by the “Fiber Tracking” module of the navigation planning software iPlan 2.6 (BrainLab, Feldkirchen, Germany) for fiber tracking, which utilizes a tensor deflection algorithm [4]. This is a type of deterministic algorithm; however, the directional trend of the fibers is also factored into the results. The direction of each step in the process was determined by weighting the previous direction by 20% and the new major eigenvector direction by 80%. Several preprocessing steps are initiated before fiber tracking can be started. This includes eddy current correction and the calculation of the apparent diffusion coefficient and fractional anisotropy (FA) maps. The calculated FA maps are then registered to the anatomic datasets (i.e., MPRAGE data) by a semi- automatic rigid registration. The following settings were utilized before performing FT: (1) the FA threshold, 0.2; (2) the minimum fiber length, 50 mm; (3) the angulations threshold, 30°; and (4) the step size, 1/3 voxel size. The tracking process was accomplished by either defining multiple rectangular volumes of interest (VOI) on the FA map or by registering standard anatomic datasets, as described in detail by Nimsky [4]. The first and largest VOI that was covered was the presumed course of the PT. The software analyzed and tracked all the fibers projecting from the voxels in the VOI. The second VOI was then placed in order to cover the precentral gyrus, which was located by anatomical landmarks and confirmed by fMRI. All of the fibers encased in the second VOI were retained for analysis. The third VOI was placed at the cerebral peduncle and retained all the fibers in the craniocaudal direction. The final tracts of interest were obtained after further excluding the inappropriate fibers. Thereby, a 3D object was generated by wrapping all the streamlines with a closed hull. This 3D object was interpolated and registered with the high-resolution 3D anatomical dataset, and allowed a clear depiction of the contour of the PT in each slice. Each PT was reconstructed by the first author and second author at the same time, both of whom were blind to the patients' clinical findings. The consistency between FT results of the two operators was evaluated by calculating the κ value according to the method reported by Wakana et al [9]. If the κ value was less than 0.7, the patient was excluded, since this situation indicates that the PT was affected excessively by the tumor or edema and the FT result might not reflect the exact PT position.

### Classifying the Location of the PT and Tumor

**Figure 1:**
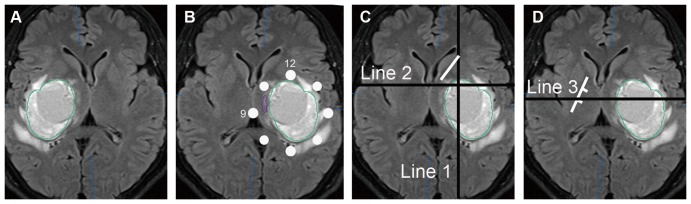
Demonstrating the method of determining the pyramidal tract position and how to draw three lines for classifying the tumors. **A**. An axial T2 FLAIR image crossing the foramen of Monro. A tumor is shown at the right basal ganglion. **B**. The tumor was mirrored to the left. The white points around the tumor indicate the o'clock scale with the 12 o'clock ahead and nine o'clock on the medial side. The PT, depicted by the purple line, was located between 12 and nine o'clock. **C**. The short, white line indicates the anterior limb of the internal capsule. The vertical, black line indicates line 1, which crossed the white line's vertex point. The horizontal black line was line 2, which crossed the foramen of Monro. **D**. The short, white line indicates the posterior limb of the internal capsule, which was divided equally into three parts. The horizontal black line indicates line 3, which crossed the marker between the middle and lateral thirds of the white line.

**Figure 2:**
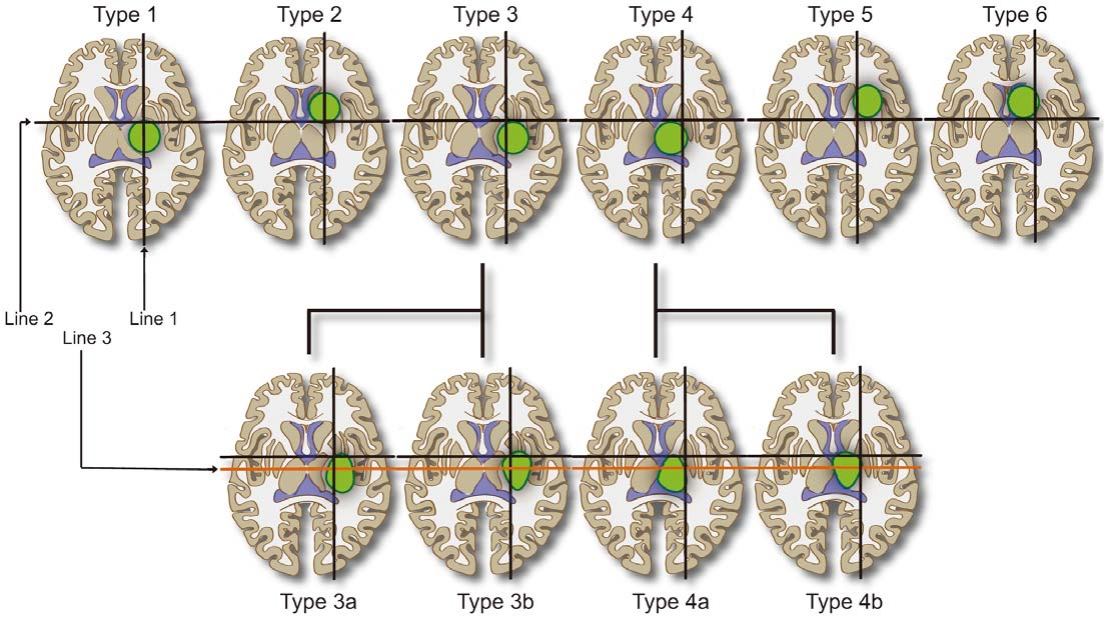
Tumors of the thalamus and basal ganglion were categorized into eight types according to three lines. From left to right: type 1 was situated below line 2 and type 2 above it, with their center lines nearly overlapping line 1. Type 3 was located in the inferior lateral region. Type 4 was located in the inferior medial region. Type 5 was in the upper lateral region. Type 6 was in the upper medial region. Type 3 and type 4 were further divided into two subgroups respectively. The main parts of tumors of type 3a or type 4a were above line 3, while type 3b and 4b were below line 3.

The location of the PT was investigated at the axial slice crossing the foramen of Monro. In order to avoid confusion and facilitate the description, all the tumors on the right side were mirrored to the left side, so that the relative position between the PT and tumor would not change in these mirrored images (Figures 1a and 1b). The area surrounding the tumor was divided into eight parts and, referring to the face of a clock, with 12 o'clock above and nine o'clock on the medial side. Relative position of the PT was classified and recorded as 12 o'clock, three o'clock, six o'clock, and nine o'clock or between two of the positions on the clock face (Figure 1b). In order to describe the tumor position, three lines were drawn manually to define the position in the basal ganglion area at the same axial slices. These three lines could be easily drawn even if the posterior limb of the internal capsule could not be identified. The first line was vertical, crossing the vertex point of the anterior limb of the internal capsule. The second line was horizontal, crossing the center of the foramen of Monro (Figure 1c). Tumors were grouped into six types, according to these two lines (Figure 2). Type 1 indicated that the main part of the tumor was below the second line, with its center line nearly overlapping the first line. Type 2 tumors had the same center line but with the main part of the tumor above the first line. Some large tumors occupying nearly the entire upper half of the basal ganglion, i.e., from the wall of the lateral ventricle to the insular cortex, were also classified as type 2. The vertical and horizontal lines crossed and formed four regions. Type 3 tumors were mainly located in the inferior lateral region. Type 4 tumors were located in the inferior medial region. Type 5 tumors were in the upper lateral region. Type 6 tumors were in the upper medial region. It must be mentioned that two additional rules were utilized during classification of the tumors. First, most tumors did not solely occupy one region, so the tumor type was judged according to the major part of the tumor. Second, the enhanced parts of some tumors occupied only small portions of the entire boundaries within abnormal signal areas in MR images. In these situations, the shape of the brain tissue with an abnormal signal was considered when classifying the tumor, since the origins of the tumors were not always related to the location of enhanced parts.

A third line was drawn for further classification of type 3 and type 4 tumors. The third line was parallel to the second line and crossed the joint of the middle and lateral thirds of the posterior limb of the internal capsule in the normal hemisphere (Figure 1d). Based on this line, type 3 and type 4 tumors were further divided into two subgroups. The main parts of type 3a or type 4a tumors were above the third line. In contrast, the majority of type 3b and 4b tumors were below the third line (Figure 2). The eight types of tumor are presented in Figure 2. Correlation analysis was performed between the position of the PTs and the tumors.

## Results

In total, there were 61 patients with thalamic or basal ganglia tumors in our hospital from March 2009 to March 2011. Two patient were excluded because the consistency between FT results of the two operators was below the standard (κ>0.7). In the first patient, the fibers of the whole internal capsule were severely affected by the tumor and surrounding edema. The fibers of the PT can only be traced with FA threshold of 0.1 and nearly all the streamlines depicted by the software interrupted before reaching the precentral gyrus. In addition, the lower FA (0.1) threshold made the streamlines increased dramatically. But without the help of precentral gyrus and clearly surrounding anatomical structures, identifying the streamlines belonging to PT became subjective. This made the consistency between the two operators much lower. In the second patients, the PT was dislocated obviously by the ganglion tumor and made the PT formulate a narrow angle besides the tumor. This made the software couldn't depict the whole course of the PT, leading to low consistency between the two operators. At last, 59 patients (26 women and 33 men; mean age, 30 years; age range, 11–67 years) were enrolled for analysis. In these patients, the PT were successfully constructed with good consistency between two operators.

In the 59 patients harboring tumors in the basal ganglion or thalamic region, six (10.17%) patients presented with type 1 tumors, six (10.17%) with type 2, seven (11.86%) with type 3a, five (8.47%) with type 3b, 17 (28.81%) with type 4a, six (10.17%) with type 4b, three (5.08%) with type 5, and nine (15.25%) with type 6.

**Figure 3:**
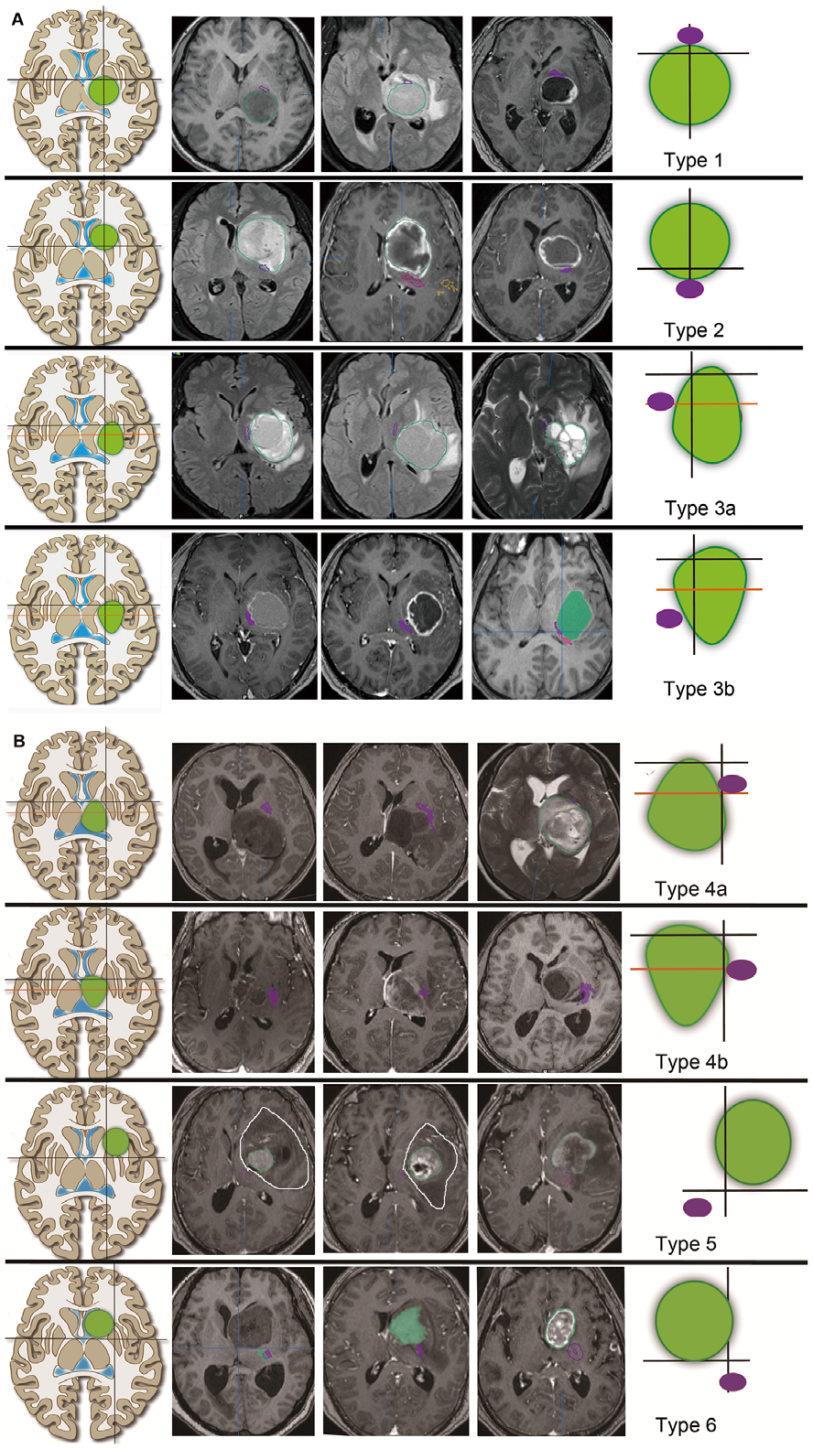
Three typical tumors and corresponding PT positions are shown for each type of tumor. **A**. Type 1 to type 3b tumors. **B**. Type 4a to type 6 tumors. The left column lists the tumor types. The right column demonstrates the relative positions of the PTs relative to each type of tumor. The green circles indicate tumors. The purple circles indicate the PT location. The middle three columns are typical cases. The PT positions are depicted by the purple line. Certain tumor boundaries are depicted by the green lines or green color, which indicate the enhanced part of the tumor. In the first and second case of type 5 tumor, the white lines show the entire brain with abnormal signals, which were much larger than the enhanced portions. These two tumor types were judged according to the shapes of the white lines, which were mainly above line 2.

The PTs were constructed successfully in all 59 patients. In the six patients with type 1 tumors, all PTs were located at the 12 o'clock position. However, in the six patients with type 2 tumors, the PTs were located at the six o'clock position. Tumors in 12 patients were classified as type 3. The PTs of these patients all were situated on the medial side of the tumors. Specifically, in the seven patients with type 3a tumors, the PTs were between nine and 12 o'clock. In contrast, in the five patients harboring type 3b tumors, the PTs all were located between six and nine o'clock. There were 23 patients harboring type 4 tumors. In these patients, the PTs were situated on the lateral side of tumor, between 12 and three o'clock in 17 patients with type 4a tumors and at three o'clock in five patients with type 4b tumors. In the three patients with type 5 tumors, the PTs were located between six and nine o'clock. In the nine patients with type 6 tumors, the PTs were between three and six o'clock. Figure 3 demonstrates the typical relative positions between the PTs and tumors in 24 patients with different types of tumor.

The majority of body of the tumor laid on the lateral side of line 1 in 15 (25.42%) patients (type 3 and type 5). In these 15 patients, the position of the PTs surrounded the medial half of the tumor. On the contrary, the PTs surrounded the lateral half of the tumor when the majority of the tumor body lay on the medial side of line 1 in another 32 (54.23%) patients (type 4 and type 6). The main body of the tumors were above line 2 in 18 (30.5%) patients (type 2, type 5 and type 6), and the PTs all were below the tumor's lowest point. Except for patients with type 1, the tumors were mainly located below line 3 in 24 (40.68%) patients (type 3a and type 4a) and the PTs were distributed around the upper half of the tumor in these patients. The majority of the tumor body was situated between line 2 and line 3 in 11 patients (type 3b and type 4b) and the PTs of these patients surrounded the lower half of the tumor. In the six patients with type 1 tumors, four were mainly below line 3, while another two were situated above this line. Hence, the rule regarding line 3 did not consistently apply to type 1 tumors.

## Discussion

The position of the PTs is one of the most important pieces of information for formulating a surgical strategy for thalamic or basal ganglia tumors [6]. Various surgical approaches have been described in the literature, and these include: transcortical transventricular approach, anterior interhemispheric transcallosal approach, contralateral infratentorial supracerebellar approach, posterior interhemispheric parasplenial approach, and transsylvian transinsular approach [1]–[3], [10]. These approaches reach the tumor from different directions, i.e., anterior, lateral and posterior to the tumor. Meanwhile, the PT can be displaced by the tumor in any direction [6], [11]. Therefore, it is very important to know where the PT is actually located before choosing the appropriate approach. For example, the surgeon must be certain that the PT is not on the lateral side of the tumor when planning the transsylvian transinsular approach. In addition, the position of the PT must be referred to during the resection of the tumor. The surgical procedures should be slower and more careful when approaching the PT, in order to avoid accidental injuries. Occasionally, this may require intentional incomplete resection of the tumor near the PT to preserve motor function [4], [5]. Prior to the development of DTI and FT techniques, it was only possible to estimate PT position according to the anatomical landmarks surrounding the tumor [6]. This continues to be a concern, since DTI and FT are still not widely used except in some major medical centers. The results of estimation are not always correct, especially when the anatomical structures cannot be identified by CT or MR images owing to tumor cell infiltration or cerebral edema [12], [13].

By using DTI-based FT, we successfully constructed all the PTs in this group of patients. The accuracy of this technique is still controversial in the literature [14]–[16], because of the limitations inherent in DTI and FT [14], [17], [18]. Some authors have demonstrated that this method cannot precisely reflect the true boundary of the PT, especially near the tumor [19],but others have confirmed that DTI-based FT can reflect the course of the fiber tracts accurately [14]. The densely packed pattern of the PT at the internal capsule was the most suitable location for the deterministic FT algorithm [20] and patients were excluded if their PT was affected excessively by the tumor or edema. On the other hand, our DTI sequence had been proved to have minimized eddy-current artifacts [4]. We routinely performed eddy current correction by using the iplan2.6 before processing of the DTI data. We did not perform the EPI distortion correction before fiber tracking, because of the limitation of the software. Even so, this shouldn't affect the accuracy of PT position in the study. The EPI distortion is more obvious when approaching the cortex and brain stem [4]. In the central region of the brain, this distortion was not obvious [4]. So, the position of the PT in this study should be reliable.

By analyzing the results of DTI-based FT, we found that three lines could help in determining the position of the PT relative to the thalamic or basal ganglia tumors, even when the tumors were large and the anatomical landmarks could not be determined. Line 1 was vertical and crossed the vertex point of the anterior limbs. Based on this line, we confirmed that the PT was on the lateral or medial side of the tumor. Line 2 and line 3 were horizontal and crossed the foramen of Monro or the joint of the middle and lateral thirds of the posterior limbs. These two lines could help in determining whether the PT followed the upper half or lower half of the tumor. As a result, tumors could be classified into eight types based on these three lines. Additionally, we further confirmed that PT position in every type of tumor had a relatively fixed pattern that appeared to be consistent. The possible reasons for this phenomenon were the PT location in the posterior limbs and special anatomical features of basal ganglion.

Historically, it was believed that the PT was located at the anterior portion of the posterior limb of the internal capsule with thalamic or basal ganglia tumors. The opinion that the PT was located at the posterior portion of the posterior limb of the internal capsule, however, seems to prevail in current research studies [21], [22]. Kim et al. confirmed, using combined fMRI/DTI and FT methods, that the typical position of the PT is at 64.27% of the posterior limb of the internal capsule [21]. This result has also been confirmed by studies using myelin stain techniques [23], [24]. We found that PT location is near the joint of the middle and lateral thirds of the posterior limb, and line 3 was designed according to this finding. The vertical line crossing the vertex point of the anterior limb intersects with posterior limbs approximately at the PT location, so line 1 was drawn to reflect this. Line 2 crossed the foramen of Monro and was actually the boundary between the anterior and posterior limbs [25]. According to these three lines, we could determine the relative position between the site of origin of the tumor and the PT. Another possible reason that the PT and tumor had a relatively fixed and consistent relationship was the anatomical features of the basal ganglion. The fibers in the internal capsule are densely packed between the thalamus, caudate nucleus and the lenticular nucleus [20]. This characteristic means the internal capsule can be displaced only and cannot be encased by the tumor [26]. Hence, the deformation of white matter by tumors with different origins would have characteristic features and demonstrated fixed patterns.

In the two patients with thalamic or basal ganglia tumors, the fiber tracking results of the PT had low consistency between the two operators. One was because the PT was severely affected by the tumor and surrounding edema. The difference of fiber tracking result of the two operators in this patient lied in the boundary of the PT. But their positions relative to tumor were consistent. The other was because the PT was dislocated obviously and formulated a narrow angle. In this patient, the positions of tracking result between the two operators were different in the axial slice crossing the foramen of Monro. The gap was about 4 mm. Even so, the relative positions between the two tracking results and tumor were consistent. These two cases still demonstrated the intrinsic characters of the algorithm of tensor calculation and fiber tracking. But, the course of the fiber tracts accurately, especially for the densely packed PT fibers, should be accurate [14], though the boundary of the tracking results might have variations. Hence, these two cases should have not influence to the conclusion, even if they were enrolled in the study. There are some limitations in our study. First, the sample size was small, especially for some types of tumors, for example, type 5 (3 cases), type 1 (6 cases) and type 2 (6 cases). Further studies with a larger sample size are still needed. Second, we did not describe data verifying the accuracy of the results of the DTI-based FT, such as surgical results, in this group of patients. Although we discussed accuracy and concluded that tract courses revealed by DTI-based FT were reliable based on the data, verification of the results is still needed. Data of surgical results still need to be analyzed and will be reported in the future. The preliminary data supported the accuracy of our FT results for determining the location of the PTs.

## Conclusions

Tumors in the thalamus and basal ganglion can be classified into eight types according to surrounding anatomical landmarks. The PT location in each type of tumor was characteristic and consistent. These results may aid surgeons in determining PT location preoperatively.
